# Autoimmune Disease in Turner Syndrome in Sweden: An up to 25 Years’ Controlled Follow-up Study

**DOI:** 10.1210/clinem/dgad566

**Published:** 2023-09-27

**Authors:** Sabine Naessén, Malin Eliasson, Kerstin Berntorp, Margareta Kitlinski, Penelope Trimpou, Emily Amundson, Sofia Thunström, Bertil Ekman, Jeanette Wahlberg, Anders Karlsson, Magnus Isaksson, Ingrid Bergström, Carina Levelind, Inger Bryman, Kerstin Landin-Wilhelmsen

**Affiliations:** Department of Women’s and Children’s Health, Karolinska Institutet, 171 77 Stockholm, Sweden; Academic Primary Health Care Centre, 117 63 Stockholm, Sweden; Primary Health Care, 442 34 Kungälv, Sweden; Genomics, Diabetes and Endocrinology Research Unit, Department of Clinical Sciences Malmö, Lund University, 222 42 Malmö, Sweden; Department of Endocrinology, Skåne University Hospital, 214 28 Malmö, Sweden; Department of Reproductive Medicine, Skåne University Hospital, 214 28 Malmö, Sweden; Section of Endocrinology, Sahlgrenska University Hospital, 413 45 Gothenburg, Sweden; Department of Internal Medicine and Clinical Nutrition, Institute of Medicine, Sahlgrenska Academy, University of Gothenburg, 413 45 Gothenburg, Sweden; Department of Internal Medicine and Clinical Nutrition, Institute of Medicine, Sahlgrenska Academy, University of Gothenburg, 413 45 Gothenburg, Sweden; Department of Respiratory Medicine, Sahlgrenska University Hospital, 413 45 Gothenburg, Sweden; Department of Internal Medicine and Clinical Nutrition, Institute of Medicine, Sahlgrenska Academy, University of Gothenburg, 413 45 Gothenburg, Sweden; Department of Clinical Genetics and Genomics, Sahlgrenska University Hospital, 413 45 Gothenburg, Sweden; Department of Endocrinology, Linköping University Hospital, 581 85 Linköping, Sweden; Department of Medicine, Örebro University Hospital, 701 85 Örebro, Sweden; School of Medical Sciences, Faculty of Medicine and Health, Örebro University, 701 12 Örebro, Sweden; Department of Medical Sciences, Uppsala University, Uppsala University Hospital, 751 85 Uppsala, Sweden; Department of Medical Sciences, Uppsala University, Uppsala University Hospital, 751 85 Uppsala, Sweden; Department of Clinical Science, Intervention and Technology, Karolinska Institutet, 171 77 Solna, Sweden; Department of Women’s and Children’s Health, Karolinska Institutet, 171 77 Stockholm, Sweden; Department of Obstetrics and Gynecology, Institute of Clinical Sciences, Sahlgrenska Academy at the University of Gothenburg, 413 45 Gothenburg, Sweden; Section of Endocrinology, Sahlgrenska University Hospital, 413 45 Gothenburg, Sweden; Department of Internal Medicine and Clinical Nutrition, Institute of Medicine, Sahlgrenska Academy, University of Gothenburg, 413 45 Gothenburg, Sweden

**Keywords:** Turner syndrome, hypothyroidism, autoimmunity, vitamin B_12_ deficiency, celiac disease

## Abstract

**Context:**

Turner syndrome (TS) is the most common chromosomal aberration in women; it is the result of structural or numeric abnormalities in the X chromosome. Autoimmune hypothyroidism has been recognized as one of the more prominent disorders associated with TS.

**Objective:**

This work aimed to study the prevalence of autoimmune diseases in TS.

**Methods:**

A cross-sectional, longitudinal, 25-year follow-up study was conducted of patients from adult Turner centers at the University Hospitals, Sweden. During 1994 to 2020, a total of 503 women aged 16 to 71 years with TS were evaluated consecutively every fifth year according to national guidelines. A random population sample of women, n = 401, aged 25 to 44 years, from the World Health Organization Monitoring of Trends and Determinants for Cardiovascular Disease (MONICA) project served as controls. Serum thyrotropin, free thyroxine, vitamin B_12_, antithyroid peroxidase (anti-TPO), and antitransglutaminase antibodies were measured.

**Results:**

Mean follow-up time (years) was 16 ± 7 for patients and 13 ± 1 for controls. From study start, the prevalence increased in TS for hypothyroidism 40% to 58%, vitamin B_12_ deficiency 5% to 12%, celiac disease 4% to 7%, positive anti-TPO 26% to 41%, and antitransglutaminase antibodies 6% to 8% (*P* < .0001 vs controls). Type 1 diabetes and Addison disease were rare. The only interrelationship was between hypothyroidism and vitamin B_12_ deficiency, both in TS and controls. No association between autoimmune disease and karyotype, antecedent growth hormone treatment, or ongoing estrogen hormone replacement, was seen in TS.

**Conclusion:**

In women with TS up to older than 80 years, more than half developed hypothyroidism, mainly autoimmune, during follow-up. Awareness of vitamin B_12_ deficiency and celiac disease throughout life is also recommended in women with TS.

Turner syndrome (TS) is among the most common chromosomal abnormalities resulting from structural or numeric abnormalities in the X chromosome with a prevalence of 1 in 2500 live-born baby girls (1, 2). The phenotype includes female sex, short stature, and primary ovarian failure; there is an association between TS and increased morbidity, mortality, and decreased life expectancy (3, 4).

Autoimmune thyroid diseases are the most prevalent organ-specific state, affecting 2% to 5% of the TS population, and the occurrence of thyroid peroxidase antibodies (anti-TPO) indicates the presence of autoimmune thyroid disease (5). However, the underlying immunopathogenic mechanism remains partially unexplained (6). Individuals with chromosomopathies seem to have autoimmune diseases more frequently than does the general population, probably due to the process of X chromosome inactivation, wherein 1 of the 2 X chromosomes undergoes inactivation or transcriptional silencing during early embryonic development (7). A close association is shown between TS and the presence of autoantibodies and autoimmune diseases, especially thyroid disease (8-12), and the risk is estimated to be approximately twice as high as in the general female population (10, 13). Nevertheless, the association between Graves disease and TS seems less frequent than expected (10, 14).

Some studies have described an increased risk of type 1 diabetes mellitus in women with TS (3, 15). Treatment with growth hormone (GH) in girls with TS might affect glucose metabolism and increase insulin resistance. However, most women who develop type 2 diabetes during GH treatment seem to have preexisting risk factors for impairment of glucose homeostasis (15).

Regarding vitamin B_12_ levels, deficiency is found based on low serum levels among TS in a previous survey of autoimmunity comorbidity (16).

Another autoimmune state is celiac disease, with a prevalence of about 4% to 5% in TS (17). A Swedish nationwide case-control study showed a 3-fold increased risk for women with TS to develop celiac disease (18). However, the pathogenesis of coexistent autoimmune thyroid disease and celiac disease is unknown, and no controlled studies of the mentioned diseases are at hand.

We have previously reported on the 5-year incidence and prevalence of hypothyroidism in TS (9). The present study is nationwide, including around 500 women with TS who were followed for up to 25 years, extending the study to include other autoimmune disorders such as vitamin B_12_ deficiency, type 1 diabetes, and celiac disease in ages above 80 years.

The aim of this study was to determine if the autoimmune propensity increased with time, whether autoimmune thyroiditis covaried with the other autoimmune conditions, and if any associations existed between autoimmunity and karyotypes. The hypotheses were that the autoimmune propensity increased with time more rapidly in women with TS than in women in general, and that one autoimmune disease would covariate with another similarly in all women irrespective of TS.

## Materials and Methods

### Patients

A cross-sectional and a longitudinal study up to 25 years was performed. All women had a diagnosis of TS, established through chromosome analysis (International Classification of Diseases, tenth revision codes Q96.0-Q96.9), and they were recruited from the departments of internal medicine, endocrinology, and obstetrics and gynecology in all university hospitals in Sweden. Most of the patients came via transition from the pediatric clinics, others came through outpatient gynecology settings and/or by referral from general practitioner due to amenorrhea for further investigation.

Data were collected during the period of 1994 to 2020, for n = 503 women, aged 16 to 71 years. The follow-up time was mean 15.6 years (range, 1-25 years).

### Control Individuals

The women with TS were compared with a random population sample of women aged 25 to 64 years, n = 870, from the World Health Organization (WHO) Monitoring of Trends and Determinants for Cardiovascular Disease (MONICA) study 1995, Gothenburg, Sweden, as a part of a worldwide collaboration in 38 countries (19). Serum hormone concentrations were analyzed in every fourth control woman between ages 25 and 34 and 35 and 44 years, and in all aged 45 to 54 and 55 to 64 years, (n = 401). They were reexamined 13.0 years later in 2008, (n = 318), with a 68% participation rate (20). Comparison of anthropometrical and biochemical data on age groups aged 25 to 34 and 35 to 44 years and for all women aged 25 to 64 years, n = 871, as well as at follow-up, are presented in Table 1. The mean follow-up time was 13 ± 1 years for the control group.

**Table 1.** Baseline anthropometric, medical and biochemical data for women with Turner syndrome and women from a population sample, the World Health Organization Monitoring of Trends and Determinants for Cardiovascular Disease (WHO MONICA) study

Variable	Turner (n = 503)	WHO MONICA (n = 401)	*P*	Difference between groups Mean (95% CI)
**Age,** mean (SD), y Median (min; max) No.	27.6 (11.5) 23 (16; 71) n = 503	35.5 (5.7) 36 (25; 45) n = 399	<.0001	−7.83 (−8.99 to −6.68)
**Height,** mean (SD), cm Median (min; max) No.	153.6 (7.2) 154 (122; 180) n = 492	166.3 (6.6) 167 (147; 182) n = 401	<.0001	−12.7 (−13.6 to −11.8)
**Body weight,** mean (SD), kg Median (min; max) No.	58.9 (11.9) 57 (30; 122.5) n = 487	65.2 (10.2) 63.4 (38.8; 110.5) n = 401	<.0001	−6.28 (−7.74 to −4.81)
**BMI,** mean (SD) Median (min; max) No.	25.1 (5.7) 24 (16.6; 102) n = 490	23.6 (3.4) 22.9 (16.8; 41.1) n = 401	<.0001	1.56 (0.95 to 2.16)
**Waist,** mean (SD), cm Median (min; max) No.	78.4 (10.6) 76 (59; 121) n = 250	76.1 (10.2) 75 (0; 113) n = 401	.032	2.32 (0.69 to 3.96)
**Hip,** mean (SD), cm Median (min; max) Number	95.7 (9.2) 95 (75; 127) n = 248	97.2 (9.8) 97 (0; 125) n = 401	.0008	−1.52 (−3.04 to 0.00)
**Waist/hip ratio,** mean (SD) Median (min; max) No.	0.813 (0.066) 0.81 (0.62; 1.14) n = 248	0.782 (0.054) 0.776 (0.663; 1.018) n = 399	<.0001	0.031 (0.021 to 0.040)
**Levothyroxine treatment,** n (%)	168/503 (33.4%)	8/401 (2.0%)	<.0001	−31.4 (−36.0 to −26.8)
**Newly detected hypothyroidism,** n (%)	37/503 (7.4%)	0/401 (.0%)	<.0001	−7.4 (−9.9 to −4.9)
**S-free T4,** means (SD), pmol/L Median (min; max) No.	15.8 (4.2) 15.3 (3; 30) n = 370	14.9 (2.5) 14.7 (7.7; 24.3) n = 96	.15	0.865 (0.198 to 1.533)
**S-TSH,** mean (SD), mU/L Median (min; max) No.	3.18 (7.78) 2.5 (0.01; 158) n = 434	1.10 (.63) 0.98 (0.01; 3.69) n = 96	<.0001	2.08 (1.34 to 2.83)
**Vitamin B_12_ supplementation,** n (%)	21/503 (4.2%)	1/401 (0.2%)	<.0001	−3.9 (−6.0 to −1.9)
**S-vitamin B_12_,** mean (SD), pmol/L Median (min; max) No.	307.1 (127.5) 288 (37; 990) n = 262	N/A	N/A	
**Newly detected vitamin B_12_ deficiency,** n (%)	2/503 (0.4%)	0/401 (0.0%)	.51	−0.4 (−1.2 to 0.4)
**Follow-up,** mean (SD), y Median (min; max) No.	15.6 (7.1) 16 (1; 25) n = 381	12.9 (0.6) 13 (12; 14) n = 70	.0004	2.66 (1.92 to 3.39)

Mean levels with SD and median with minimum; maximum levels are shown for all numerical variables. n = number of participants and CI are shown for each variable. Number (n) and percentage (%) are shown for categorical variables.

Abbreviations: BMI, body mass index; N/A, not available; S, serum; S-TSH, serum thyrotropin; T4, thyroxine.

### Examinations

All women with TS had blood pressure measurements, clinical checkups, and thyroid function tests every fifth year according to the Swedish Turner Academy recommendations by an endocrinologist, or by a gynecologist at the Turner centers at all university hospitals in Sweden (21). Height and body weight were measured in light clothes and without shoes. Body mass index (BMI) = weight in kg divided by the square of height in meters. Waist and hip circumference were measured over the umbilicus and the widest part of the hip, respectively, and the waist/hip ratio calculated.

### Medications

Medications, coded according to the Anatomical Therapeutic Chemical code, were registered at all examinations at start and follow-up. Supplementation with levothyroxine = H03AA, vitamin B_12_ = B03BA, calcium/vitamin D = A12AX, insulin = A10A, and estrogen hormone replacement, menopausal hormone therapy (MHT) = G03 were checked. The same medications were retrieved for the women in the WHO MONICA population (19). For the women with TS, previous GH treatment was recorded.

### Biochemical Analyses

Fasting blood samples were drawn in similar ways in patients and control participants at baseline and at the follow-up examination. Samples in menstruating women were collected on cycle day 7 to 9. After centrifugation, serum and plasma aliquots were frozen in 1-mL glass ampoules and stored at −70 °C until analysis, which took place within 1 year for all variables. The analyses were performed with the same analytical reagents at the accredited laboratory for clinical chemistry at the university hospitals. Serum free thyroxine (T4; reference range, 12-22 pmol/L); serum thyrotropin (TSH; reference range, 0.30-4.2 mU/L); TPO antibody concentration (reference range, < 60 kU/L); serum vitamin B_12_ (reference range, 140-500 pmol/L); serum folate (reference range, 6-35 nmol/L); serum 25-hydroxyvitamin D, (25(OH)D; reference range, ≥ 25 nmol/L); and celiac disease, antitransglutaminase antibody concentration including immunoglobulin (Ig)A transglutaminase, IgA transglutaminase/endomysium, IgG deamidated gliadin, IgA gliadin, IgG gliadin, and IgA antiendomysium (reference range, positive or negative) were checked. All data were gathered in Microsoft Excel documents.

#### Definitions for the different states

Hypothyroidism: supplementation with levothyroxine or a serum TSH concentration greater than 4.2 mU/L and/or serum free T4 less than 12 pmol/L.

Autoimmune hypothyroidism: hypothyroidism, treated or newly detected, and a TPO antibody concentration greater than 60 kU/L.

Positive antitransglutaminase antibodies: a positive titer of either IgA transglutaminase, IgA transglutaminase/endomysium, IgG deamidated gliadin, IgA gliadin, IgG gliadin, or IgA antiendomysium.

Vitamin B_12_ deficiency was present if supplementation was given and/or if a serum level of less than 140 pmol/L was found.

Vitamin D deficiency was defined as a serum 25(OH)D level of less than 25 nmol/L and insufficiency less than 50 nmol/L, respectively.

A previous or current disease of celiac disease, Addison disease, treated vitamin B_12_ deficiency, or type 1 diabetes mellitus was checked in the medical record for the women with TS and control individuals at baseline and at follow-up.

### Statistical Analysis

The first and the last (range, 1-25 years) follow-up data were used for all women. For continuous variables, mean (SD)/median (minimum; maximum)/n = is presented. For comparison between groups, the Mann-Whitney *U* test was used for continuous variables. Calculations of CI for continuous variables were based on the assumption of normality. When variances were not equal (*P* < .05), the SD was based on Satterthwaite's approximation; otherwise the SD was based on the pooled SDs. To investigate the covariation between autoimmune thyroiditis and the other autoimmune states and diseases, the Spearman rank correlation test was used. *P* values less than .05 were considered statistically significant, and not significant is marked NS.

### Ethical Considerations

This study was approved by the regional ethical review board in Stockholm for the National project Dnr Ö5-2010 and for the WHO MONICA Project, Dnr 088-06 and Dnr T282-11.

## Results

### Baseline Investigations

The baseline investigation took place in 1994 to 1995 both for women with TS and control participants. Of the TS cohort, 244 (49%) had monosomy X; 86 (17%) 45, X/46, XX mosaicism; and 133 (27%) either iso, ring, or deletion of an X chromosome or a Y chromosome fragment, of which the latter was present in 30 (6%). The karyotype could not be traced in 10 women and the TS diagnosis was based on other medical documents. Anthropometric and biochemical analysis data are given for women with TS and control individuals in Tables 1 to 3. Women with TS were shorter and had lower body weight but higher BMI and waist/hip ratio than controls (see Tables 1 and 2). Free T4 and TSH levels were higher in women with TS than control participants. Serum (S) 25(OH)D levels were similar between the groups. Of the 503 women with TS, 239 (48%) had been treated with GH and 377 (75%) had ongoing MHT. Oral contraceptives or MHT were prescribed in 18% of the population sample.

**Table 2.** Data for women with Turner syndrome in Sweden in comparison with the entire random population sample of women aged 24 to 64 years, the World Health Organization Monitoring of Trends and Determinants for Cardiovascular Disease (MONICA) study, at start (0) and at follow-up

Variable	Turner (n = 503)	MONICA (n = 871)	*P*	Difference between groups Mean (95% CI)
**Age, 0** mean (SD), y Median (min; max) No.	27.6 (11.5) 23 (16; 71) n = 503	45.7 (11.1) 46 (25; 65) n = 856	<.0001	−18.1 (−19.3 to −16.8)
**Age, Fu** mean (SD), y Median (min; max) No.	42.3 (13.8) 40 (18; 85) n = 384	63.7 (9.0) 65 (39; 78) n = 319	<.0001	−21.4 (−23.1 to −19.7)
**BMI, 0** mean (SD) Median (min; max) No.	25.1 (5.7) 24 (16.6; 102) n = 490	24.7 (4.1) 23.9 (16.8; 47.2) n = 868	.49	0.428 (−.145 to 1.000)
**BMI, Fu** mean (SD) Median (min; max) No.	25.8 (5.3) 24.8 (16; 42.6) n = 135	26.6 (5.0) 25.6 (17.2; 54.8) n = 318	.052	−0.848 (−1.882 to 0.186)
**Waist/hip ratio, 0** mean (SD) Median (min; max) No.	0.813 (0.066) 0.81 (0.62; 1.14) n = 248	0.796 (0.060) 0.791 (0.641; 1.035) n = 866	.0003	0.016 (0.007 to 0.025)
**Waist/hip ratio, Fu** mean (SD) Median (min; max) No.	0.839 (0.085) 0.825 (0.69; 1.29) n = 120	0.836 (0.077) 0.832 (0.624; 1.356) n = 314	.85	0.003 (−0.014 to 0.019)
**P-glucose, 0** mean (SD), mmol/L Median (min; max) No.	4.88 (1.53) 4.6 (3.4; 21) n = 283		N/A	
**P-glucose, Fu** mean (SD), mmol/L Median (min; max) No.	5.50 (1.33) 5.2 (3.8; 12.5) n = 116	5.20 (1.22) 5 (3.2; 14.1) n = 316	.0006	0.299 (0.033 to 0.564)
**S-free T4, 0** Mean (SD), pmol/L Median (min; max) No.	15.8 (4.2) 15.3 (3; 30) n = 370	15.2 (2.7) 14.8 (7.7; 30.4) n = 214	.26	0.576 (0.009 to 1.143)
**S-free T4, Fu** mean (SD), pmol/L Median (min; max) No.	17.3 (4.1) 17 (6; 32) n = 327	16.2 (2.8) 16 (10; 45) n = 316	<.0001	1.12 (0.57 to 1.66)
**S-TSH, mU/L, 0** mean (SD) Median (min; max) No.	3.18 (7.78) 2.5 (0.01; 158) n = 434	1.34 (1.55) 1.08 (0; 19.4) n = 214	<.0001	1.84 (1.08 to 2.60)
**S-TSH, mU/L, Fu** mean (SD) Median (min; max) No.	3.10 (6.18) 2.5 (0.01; 100) n = 292	2.41 (1.60) 2.15 (0.01; 16) n = 316	.037	0.689 (−0.044 to 1.422)
**Fu** mean (SD), y Median (min; max) No.	15.6 (7.1) 16 (1; 25) n = 381	12.7 (0.6) 13 (12; 14) n = 311	<.0001	2.85 (2.13 to 3.57)

Mean levels with SD, median with minimum; maximum levels, number (n), and CIs are shown.

Abbreviations: BMI, body mass index; Fu, follow-up; N/A, not available; P, plasma; S, serum; S-TSH, serum thyrotropin; T4, thyroxine.

**Table 3.** Data for women with Turner syndrome in Sweden in comparison with the entire random population sample of women aged 24 to 64 years, the World Health Organization Monitoring of Trends and Determinants for Cardiovascular Disease (MONICA) study, at start (0) and at follow-up

Variable	Turner (n = 503)	MONICA (n = 871)	*P*	Difference between groups Mean (95% CI)
**S-B_12_, 0** mean (SD), pmol/L Median (min; max) No.	307.1 (127.5) 288 (37; 990) n = 262		N/A	
**S-B_12_, Fu** mean (SD), pmol/L Median (min; max) No.	390.0 (206.1) 330 (147; 1470) n = 173	415.3 (211.4) 360 (130; 1470) n = 317	.030	−25.4 (−64.3 to 13.6)
**S-folate, 0** mean (SD), nmol/L Median (min; max) No.	18.6 (26.2) 14 (4.9; 370) n = 208		N/A	
**S-folate, Fu** mean (SD), nmol/L Median (min; max) No.	18.6 (9.8) 16 (6.6; 45) n = 57	21.5 (9.7) 18 (7.2; 45) n = 317	.017	−2.85 (−5.60 to −0.11)
**S-25(OH)D, 0** mean (SD), nmol/L Median (min; max) No.	50.7 (22.9) 48 (5; 158) n = 144	50.0 (17.3) 48 (13; 131) n = 258	.96	0.712 (−3.596 to 5.021)
**S-25(OH)D, Fu** mean (SD), nmol/L Median (min; max) No.	67.6 (23.3) 61 (18.5; 159) n = 105	64.7 (25.3) 60.2 (16.2; 163) n = 318	.11	2.88 (−2.61 to 8.37)
**Fu** mean (SD), y Median (min; max) No.	15.6 (7.1) 16 (1; 25) n = 381	12.7 (0.6) 13 (12; 14) n = 311	<.0001	2.85 (2.13 to 3.57)

Mean levels with SD, median with minimum; maximum levels, number (n), and CIs are shown.

Abbreviations: Fu, follow-up; N/A, not available; S, serum; S-25(OH)D, serum 25-hydroxyvitamin D.

### Autoimmune Diseases

Treated hypothyroidism was present in 168 (33%) women with TS vs 8 (2%) in control individuals (*P* < .0001) (see Table 1). Elevated serum TSH values (>4.2 mU/L) were found in another 7% and 0%, respectively (see Table 1). Altogether, treated and newly detected hypothyroidism was found in 40% of women with TS and 2% of control participants (*P* < .0001). Elevated anti-TPO was found in 26% of women with TS and in 4% of control individuals (*P* < .0001). Among the women with TS who had hypothyroidism, 14% had elevated TPO compared with 1% of women with hypothyroidism in the general population sample (*P* < .0001). Two women with TS and 2 control women had had Graves disease.

Among women with TS, 4% had treated vitamin B_12_ deficiency and another 2 women with TS were newly detected, in total 5% compared to none in the control population (*P* < .0001) (see Table 1). Vitamin D deficiency (defined as S-25(OH)D < 25 nmol/L) was found in 3% of women with TS but in none of the control individuals (*P* < .05).

Positive antitransglutaminase antibodies were present in 6% of women with TS and were not measured in controls at baseline. Overt celiac disease was found in 4% of women with TS and none among control participants (*P* < .0001). Insulin treatment for type 1 diabetes was present in 1% of women with TS and 1% in control women (NS). Addison disease was not present in any women with TS or any control women. The different diseases were evenly distributed among the karyotypes in women with TS. Anti-TPO was less common, but celiac antibodies were more frequently found in TS with iso, ring, or a deletion karyotype (Table 4).

**Table 4.** Frequency of autoimmune disorders in different karyotypes in women with Turner syndrome in Sweden

	Monosomy, 45, X (n = 259)	Mosaicism, 45, X/46, XX (n = 106)	Iso, ring, deletion (n = 107)	Y fragment (n = 30)
**Hypothyroidism, n (%)**	105 (41)	42 (38)	44 (41)	14 (47)
**Elevated TPO, n (%)**	82 (32)	23 (22)	14 (13)*^b^*	6 (20)
**Vitamin B_12_ deficiency, n (%)**	10 (4)	6 (6)	6 (6)	1 (3)
**Celiac disease, n (%)**	12 (5)	4 (4)	3 (3)	0 (0)
**Positive antitransglutaminase antibodies, n (%)**	10 (4)	4 (4)	14 (13)*^a^*	2 (7)

N, number (percentage), antitransglutaminase, celiac disease antibodies.

Abbreviation: TPO, thyroid peroxidase antibodies.

^*a*^ *P* less than .01 and *^b^P* less than .001 vs monosomy. No other statistically significant differences between the karyotype groups were found.

### Follow-up Examination

Mean follow-up was 15.6 years (range, 1-25 years) for TS patients. The mean follow-up time for the random control population sample was 12.9 years (range, 12-14 years). The mean age in women with TS at follow-up was 42.3 years (range, 18-85 years). Body weight and height were lower, but BMI and waist/hip ratio were similar in women with TS and control women (see Tables 2 and 5). Serum thyroid hormones and TPO, S-25(OH)D, and plasma glucose were higher in women with TS than control women, while vitamin B_12_ and folate were similar compared to controls (see Tables 5 and 6). However, vitamin B_12_ and folate were lower in women with TS at follow-up compared to the entire control group (see Table 3).

**Table 5.** Baseline (0) and follow-up of anthropometric and biochemical data for women with Turner syndrome and women from a population sample, the World Health Organization Monitoring of Trends and Determinants for Cardiovascular Disease (MONICA) study

Variable	Turner (n = 384)	MONICA (n = 70)	*P*	Difference between groups Mean (95% CI)
**Age, 0** mean (SD), y Median (min; max) No.	26.9 (10.8) 22 (16; 64) n = 384	37.1 (5.5) 39 (26; 44) n = 70	<.0001	−10.2 (−11.9 to −8.5)
**Age, Fu** mean (SD), y Median (min; max) Number	42.3 (13.8) 40 (18; 85) n = 384	50.0 (5.5) 52 (39; 58) n = 70	<.0001	−7.71 (−9.62 to −5.80)
**Height, 0** mean (SD), cm Median (min; max) No.	153.7 (6.9) 154 (131; 176) n = 379	166.2 (6.7) 166.5 (150; 182) n = 70	<.0001	−12.5 (−14.3 to −10.8)
**Height, Fu** mean (SD), cm Median (min; max) No.	152.4 (6.9) 153 (132; 169) n = 188	165.6 (6.6) 165.1 (151.4; 180.5) n = 70	<.0001	−13.2 (−15.0 to −11.3)
**Weight, 0** mean (SD), kg Median (min; max) No.	59.0 (11.1) 57.4 (33; 99) n = 377	64.5 (10.0) 64.6 (44.8; 88.1) n = 70	<.0001	−5.44 (−8.22 to−2.65)
**Weight, Fu** mean (SD), kg Median (min; max) No.	60.3 (13.7) 57 (32; 106) n = 186	69.8 (11.5) 68.2 (46.9; 96.3) n = 70	<.0001	−9.57 (−13.20 to −5.94)
**BMI, 0** mean (SD) Median (min; max) No.	25.2 (5.9) 24.1 (17.8; 102) n = 377	23.3 (3.1) 22.8 (17.9; 33) n = 70	.0036	1.90 (0.94 to 2.85)
**BMI, Fu** mean (SD) Median (min; max) No.	25.8 (5.3) 24.8 (16; 42.6) n = 135	25.4 (3.8) 24.8 (18.7; 36.6) n = 70	≥.999	0.354 (−0.923 to 1.632)
**Waist, 0** mean (SD), cm Median (min; max) No.	78.4 (10.7) 76 (61; 110) n = 161	75.9 (8.0) 74 (63; 98) n = 70	.16	2.48 (−0.03 to 4.99)
**Waist, Fu** mean (SD), cm Median (min; max) No.	81.9 (11.6) 78.5 (64; 106.5) n = 32	86.0 (10.7) 86 (66; 115) n = 70	.051	−4.15 (−8.81 to 0.52)
**Hip, 0** mean (SD), cm Median (min; max) No.	95.5 (8.9) 95 (75; 122) n = 159	97.2 (6.6) 98 (82; 113) n = 70	.045	−1.77 (−3.85 to 0.32)
**Hip, Fu** mean (SD), cm Median (min; max) No.	96.5 (9.4) 95 (74; 112) n = 22	103.2 (8.8) 104 (86; 126) n = 70	.0071	−6.69 (−11.01 to −2.36)
**Waist/hip ratio, 0** mean (SD) Median (min; max) No.	0.812 (0.067) 0.81 (0.62; 1.14) n = 159	0.780 (.051) 0.773 (0.68; 0.904) n = 70	.0002	0.032 (0.017 to 0.048)
**Waist/hip ratio, Fu** mean (SD) Median (min; max) No.	0.839 (0.085) 0.825 (0.69; 1.29) n = 120	0.831 (0.064) 0.833 (0.705; 0.971) n = 68	.89	0.008 (−0.014 to 0.029)

Mean levels with SD, median with minimum; maximum levels, number (n), and CIs are shown.

Abbreviations: BMI, body mass index; Fu, follow-up.

**Table 6.** Baseline (0) and follow-up of anthropometric and biochemical data for women with Turner syndrome and women from a population sample, the World Health Organization Monitoring of Trends and Determinants for Cardiovascular Disease (MONICA) study

Variable	Turner (n = 384)	MONICA (n = 70)	*P*	Difference between groups Mean (95% CI)
**Free T4, 0** mean (SD), pmol/L Median (min; max) No.	15.3 (4.3) 15 (3; 30) n = 289	14.7 (2.6) 14.4 (10; 24.3) n = 58	.62	0.557 (−0.277 to 1.391)
**Free T4, Fu** mean (SD), pmol/L Median (min; max) No.	17.3 (4.1) 17 (6; 32) n = 327	16.1 (2.0) 16 (12; 22) n = 69	.0047	1.22 (0.56 to 1.88)
**TSH, 0** mean (SD), mU/L Median (min; max) No.	3.35 (8.82) 2.5 (0.01; 158) n = 333	1.12 (0.49) 1.06 (0.06; 2.54) n = 58	<.0001	2.24 (1.28 to 3.19)
**TSH, Fu** mean (SD), mU/L Median (min; max) No.	3.10 (6.18) 2.5 (0.01; 100) n = 292	2.11 (1.24) 2 (0.05; 6.8) n = 69	.0066	0.997 (0.227 to 1.767)
**Vitamin B_12_, 0** mean (SD), pmol/L Median (min; max) No.	305.0 (123.6) 281 (96; 990) n = 177		N/A	
**Vitamin B_12_, Fu** mean (SD), pmol/L Median (min; max) No.	390.0 (206.1) 330 (147; 1470) n = 173	385.5 (148.8) 350 (160; 800) n = 69	.34	4.46 (−42.51 to 51.44)
**Folate, 0** mean (SD), nmol/L Median (min; max) No.	19.9 (31.4) 14 (4.9; 370) n = 141		N/A	
**Folate, Fu** Mean (SD), nmol/L Median (min; max) No.	18.6 (9.8) 16 (6.6; 45) n = 57	20.4 (9.2) 18 (7.2; 45) n = 69	.13	−1.79 (−5.15 to 1.57)
**25(OH)D, 0** mean (SD), nmol/L Median (min; max) No.	48.6 (18.8) 46 (14.8; 96) n = 101	57.1 (18.9) 54 (26; 85) n = 19	.077	−8.49 (−17.80 to 0.83)
**25(OH)D, Fu** mean (SD), nmol/L Median (min; max) No.	67.6 (23.3) 61 (18.5; 159) n = 105	56.9 (18.5) 54.2 (16.2; 94.6) n = 70	.0017	10.8 (4.5 to 17.0)
**Glucose, 0** mean (SD), mmol/L Median (min; max) No.	4.76 (1.27) 4.5 (3.4; 13.9) n = 191	N/A		
**Glucose, Fu** mean (SD), mmol/L Median (min; max) No.	5.50 (1.33) 5.2 (3.8; 12.5) n = 116	4.97 (1.12) 4.8 (3.7; 13) n = 68	<.0001	0.533 (0.155 to 0.911)
**Fu,** mean (SD), y Median (min; max) No.	15.6 (7.1) 16 (1; 25) n = 381	12.9 (0.6) 13 (12; 14) n = 70	.0004	2.66 (1.92 to 3.39)

For comparison between groups, the Mann-Whitney *U* test was used for continuous variables. Calculations of CIs for continuous variables are based on the assumption of normality. When variances are not equal (*P*<.05) the SD is based on Satterthwaite's approximation; otherwise, the SD is based on the pooled SDs.

Mean levels with SD, median with minimum; maximum levels, number (n), and CIs are shown.

Abbreviations: 25(OH)D, 25-hydroxyvitamin D; Fu, follow-up; N/A, not available; T4, thyroxine; TSH, thyrotropin.

**Figure 1.**
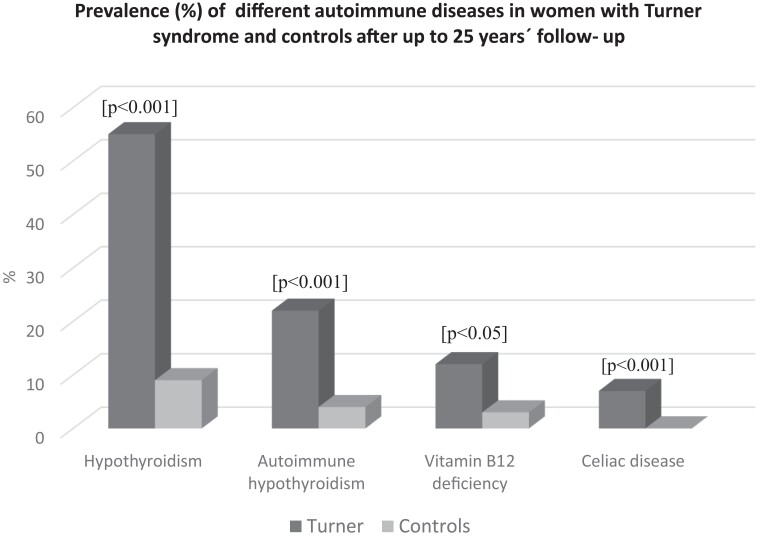
Prevalence (%) of different autoimmune diseases in women with Turner syndrome and population-based controls, the WHO MONICA study, after up to 25 years’ follow-up. Significances for tests between patients and controls are given above each paired bar.

Hypothyroidism in TS had increased from 40% to 58% vs 2% to 9% of controls at this time and at a mean age 42 years (range, 18-85 years) and 50 years (range, 39-58 years), respectively, Fig. 1. The number of individuals with elevated TPO antibodies had increased from 26% to 41% in the TS group and from 4% to 10% in the control group during these years. S-TSH increased continuously, also within normal range, but not all women developed hypothyroidism with increasing age. Those with elevated TPO were at highest risk for doing so both in women with and without TS.

**Figure 2.**
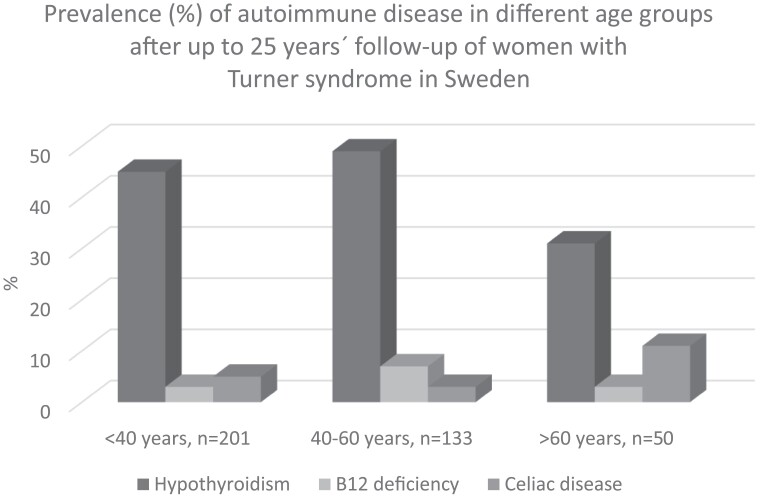
Prevalence (%) of autoimmune disease after up to 25 years’ follow-up in different age groups (years) of women with Turner syndrome in Sweden.

Treated vitamin B_12_ deficiency increased both in TS patients from 5% to 12% and in control participants from 0% to 3% at follow-up, and celiac disease had increased in women with TS from 4% to 7% (see Fig. 1). Vitamin D deficiency and diabetes mellitus were similar in TS as in controls. Calcium/vitamin D supplementation was more prevalent in TS, 6% at start and 14% at follow-up, than in the control women of similar age, of whom none required calcium/vitamin D supplementation at start or at follow-up. None of the women with TS or controls had Addison disease or newly detected diabetes mellitus at follow-up. The prevalence of the most frequent autoimmune states in TS and controls is shown in Fig. 1, and the distribution by age in TS appears in Fig. 2.

The distribution of the studied diseases did not change among the karyotypes in women with TS during the study. There were no differences in the prevalence or incidence of autoimmune states irrespective of having received GH or ongoing MHT in women with TS or in women with MHT in the population sample, respectively.

### Interrelationship Between Autoimmune Hypothyroidism and Other Autoimmune States and Diseases

Interaction between autoimmune diseases was found between hypothyroidism and vitamin B_12_ deficiency, which was seen both in women with TS and control participants (Table 7). Elevated antibodies for celiac disease, antitransglutaminase antibodies, were more commonly found together with hypothyroidism in controls (see Table 7). The women with vitamin B_12_ deficiency, treated or newly detected, were other than those with elevated celiac antibodies or celiac disease both in the TS and control groups.

**Table 7.** Spearman rank correlation test for the interrelationship (*R*) between hypothyroidism or elevated thyroid peroxidase antibody concentration or autoimmune thyroiditis and other autoimmune states and diseases in women with Turner syndrome aged 20 to 85 years and in control individuals from a randomized sample of women (World Health Organization Monitoring of Trends and Determinants for Cardiovascular Disease [MONICA] project, Gothenburg) aged 38 to 78 years

Variable	Hypothyroidism *R*	*P*	Elevated TPO *R*	*P*	Autoimmune thyroiditis *R*	*P*
**Vitamin B_12_ deficiency**	0.173*^a^* (.134*^a^*)	**.02 (.017)**	0.108 (−.027)	.164 (.638)	0.116 (−.180)	.136 (.751)
**Vitamin D insufficiency**	−0.180 (−.048)	.073 (.392)	0.040 (−.046)	.718 (.414)	−0.091 (−.031)	.406 (.582)
**Positive antitransglutaminase antibodies**	0.056 (.132*^a^*)	.502 **(.037)**	0.031 (−.029)	0716 (.625)	0.014 (−.020)	.868 (.751)
**Celiac disease**	−0.075 (−.028)	.326 (.624)	−0.034 (−.027)	.663 (.636)	−0.019 (−.018)	.811 (.750)
**T1DM**	0.001 (−)	.994 (−)	0.148 (−)	.057 (−)	0.046 (−)	.559 (−)
**Addison disease**	− (−)	− (−)	− (−)	− (−)	− (−)	− (−)

Abbreviations: T1DM, type 1 diabetes mellitus; TPO, thyroid peroxidase antibody.

^*a*^Correlation is statistically significant at the .05 level. Significant *P* values are marked in bold. *R* and *P* values are given in the table for patients and in parentheses for control individuals.

## Discussion

In ages above 80 years, every second woman with TS had developed hypothyroidism after up to 25 years’ follow-up. The overall prevalence was 3 to 5 times higher in TS than in women from the general population of different age groups. Autoimmune hypothyroidism dominated in women with TS. The result confirms previous cross-sectional studies and 5-year follow-up data in adults with TS (8-12).

This further-extended, national cohort study is unique, with similar examinations at all university hospitals in the country. Not all women with TS developed hypothyroidism but those with elevated TPO antibodies were at highest risk. It is important to keep in mind that the women with TS in the present study had their thyroid function checked regularly and, thus, frequent blood sampling could contribute to the increased detection rate. However, follow-up was present, both for women with TS and controls, and no such high prevalence for hypothyroidism was found in the control participants, though these individuals were older and the control time was shorter (see Tables 1 and 2 and Fig. 1).

Vitamin B_12_ deficiency was associated with hypothyroidism both in women with TS and control individuals but more frequently so in women with TS. This is a new finding in TS and was independent of malabsorption as in celiac disease. Only a few studies are at hand considering whether vitamin B_12_ deficiency is more common in women with TS (11, 16). In the Danish study (11), no results of vitamin B_12_ supplementation were recorded. This is the first study to report both on the treatment and newly detected cases according to blood tests, prospectively. Vitamin B_12_ deficiency was more common in those with hypothyroidism in general and not specifically in those with autoimmune hypothyroidism or with TS, respectively. Hence, annual blood test regarding thyroid function is recommended and vitamin B12 analyses at least every fifth year.

Vitamin D deficiency (S-25(OH)D < 25 nmol/L) was rare but low levels in general (insufficiency defined as S-25(OH)D ≥ 25-<50 nmol/L) have been reported in TS (1, 22). The fairly sufficient levels of S-25(OH)D in the present study might be a result of the supplementation with calcium/vitamin D due to osteoporosis to a greater extent than in the control group. Those with vitamin D deficiency among TS were not those who had celiac disease.

Celiac disease as well as positive antibodies for this disease were more frequent in TS and has been reported in children with TS (11, 18, 23). Mårild et al (18) compared 7548 women with biopsy-verified celiac disease with 34 492 women in a control group. Of the 7548 women with celiac disease, 20 had a diagnosis of TS (0.26%) compared to 21 of the 34 492 women in the control group (0.06%). The authors conclude therefore that patients with TS had a 3-fold increased risk of developing celiac disease. The figure is in harmony with the present longitudinal study. Gastroscopy was performed only when biochemical analyses indicated signs of malabsorption in the present study.

Addison disease was not present in any of the women with TS or control participants in this study, which is in line with Stenberg et al (24).

Previous studies have reported an increased prevalence of type 1 diabetes mellitus in women with TS (3, 7, 25). In this study, diabetes mellitus was rare. It would have been of interest to have tested for antibodies associated with type 1 diabetes mellitus (eg, insulin autoantibodies, islet cell cytoplasmic antibodies). BMI at follow-up was not higher in women with TS than control women and might have contributed to few cases of diabetes mellitus in general. BMI was greater than 30 in the American survey (7) and high in the Danish studies (3, 25). Vitamin B_12_ deficiency covaried with hypothyroidism irrespective of TPO antibody concentration. A similar pattern, albeit to a lesser extent, was also seen in control individuals. The studied diseases were evenly distributed among the karyotypes consistent with previous studies (10, 11). Hence, the autoimmune propensity was more frequent in TS but the interplay between the different autoimmune diseases were similar to those in women in the general population. MHT was induced in similar numbers of women with TS and control individuals with and without hypothyroidism, respectively. Therefore, it seems unlikely that MHT precipitates or induce hypothyroidism. In addition, some of the women with TS developed hypothyroidism long before puberty induction with estrogen. MHT use in postmenopausal women in the general population as a control group declined during follow-up, from 26% to less than 10% (20). This mirrors the results from the Women's Health Initiative Study (26). Thyroid hormone fluctuations are more prevalent in women than in men, as are sex hormone changes during puberty, pregnancy, and menopause (27). The influence of male and female sex hormones on thyroid hormones and hypothyroidism was studied by Schmidt et al (28), showing that men and women with hyperandrogenism as in polycystic ovary syndrome had less hypothyroidism than premenopausal and postmenopausal women and TS, respectively. This does not, however, explain the reason why hypogonadal girls with TS develop hypothyroidism even before puberty induction with estrogen was started. Furthermore, women with TS harboring a Y-fragment in the present study did not differ from other karyotypes in this aspect.

Glynn et al (29) showed that following GH treatment, free T4 concentrations declined, and free 3,5,3′-triiodothyronine levels increased in girls with TS. Comparing those who had received GH in the present study with those without, irrespective of age, did not give evidence for the hypothesis of GH-induced hypothyroidism in TS.

The studies by Bakalov et al (7) revealed a 2-fold increase in the prevalence of hypothyroidism in women with idiopathic 46, XX primary ovarian insufficiency in whom the incidence was twice that in women with TS, suggesting that androgen deficiency may be implicated in the pathogenesis of Hashimoto thyroiditis (7).

Androgen levels are reduced in women with 46, XX and primary ovarian insufficiency, and even lower in women with TS, and these low levels of androgens might be linked to an increased risk of hypothyroidism in women with ovarian insufficiency (3, 7, 30). The absence of a normal second X chromosome and the resultant haploinsufficiency for X-chromosome–related gene(s) may further increase the risk for thyroid and possibly other autoimmune disorders (7).

However, the markedly high incidence of hypothyroidism both at young age as well as in older ages in women with TS seems to be linked to the genetic syndrome per se. Of note, some women with TS had neither hypothyroidism nor elevated TPO up to age 85 years with various karyotypes in the present national survey.

### Strengths

One of the major strengths of this study was the large group of women with TS (n = 503) from all Turner centers in Sweden followed since 1994 to ages above 80 years and the randomized population sample recruited from the city census followed in parallel, the WHO MONICA, as the control group.

All patients in the study group were similarly evaluated clinically and biochemically and managed therapeutically according to the national and international guidelines established at that time (31) and updated (32). More than 30% of women with TS had reached age 45 years or older, which is a considerably high age for follow-up studies in TS. Controlled studies in TS are sparse.

### Limitations

The findings of this study need to be considered in the context of some limitations.

Serum vitamin B_12_ and celiac autoantibodies for women in the control group were not analyzed at baseline. However, serum vitamin B_12_ deficiency was detected in only one woman in the control group at the follow-up examination. Therefore, vitamin B_12_ substitution is probably a good proxy for this diagnosis.

Gastroscopy was performed only in women with TS who had positive celiac autoantibodies together with signs of malabsorption to verify a diagnosis of celiac disease. Therefore, it is hard to know if all women with positive autoantibodies have overt celiac disease. However, a recent review of more than 6000 patients with TS including serology and biopsy-based celiac disease showed a positive association between the two methods and a prevalence of celiac disease of 4% in TS (33). This is in line with the present clinical observation.

Another limitation was that women with TS had more regular contact with health care providers than women in the control group. The repeated medical checks may have led to earlier detection and treatment of disorders to a greater extent in women with TS than in control individuals between the examinations at start and follow-up. However, the biochemical data at the follow-up in control women did not indicate more than sporadic cases with undiagnosed autoimmune diseases that then were further examined and treated (Tables 2, 3, and 6). The control group was older and not completely age-matched with the study population. Nevertheless, since both groups were compared at the end of the study and most autoimmune disorders will increase in prevalence over time, the age difference favors underestimating the prevalence of autoimmune disorders in TS and hence, rather strengthens the conclusions.

In conclusion, autoimmune diseases were common in TS, irrespective of karyotype, previously GH treatment, or ongoing MHT. Every second woman with TS developed hypothyroidism during up to 25 years’ follow-up in ages older than 80. Thyroid function should be checked annually, and awareness of vitamin B_12_ deficiency and celiac disease throughout life is recommended in women with TS.

## Data Availability

The data are available by request from the corresponding author.

## Abbreviations

25(OH)D: 25-hydroxyvitamin D
GH: growth hormone
Ig: immunoglobulin
MHT: menopausal hormone therapy
MONICA: Monitoring of Trends and Determinants for Cardiovascular Disease
S: serum
T4: thyroxine
TPO: thyroid peroxidase antibody
TS: Turner syndrome
TSH: thyrotropin
WHO: World Health Organization

## References

[1] Gravholt CH . Epidemiological, endocrine and metabolic features in Turner syndrome. Eur J Endocrinol. 2004;151(6):657‐687. 15588233 10.1530/eje.0.1510657

[2] Davenport ML . Approach to the patient with Turner syndrome. J Clin Endocrinol Metab. 2010;95(4):1487‐1495. 20375216 10.1210/jc.2009-0926

[3] Gravholt CH , Juul S, Naeraa RW, Hansen J. Morbidity in Turner syndrome. J Clin Epidemiol. 1998;51(2):147‐158. 9474075 10.1016/s0895-4356(97)00237-0

[4] Stochholm K , Juul S, Juel K, Naeraa RW, Gravholt CH. Prevalence, incidence, diagnostic delay, and mortality in Turner syndrome. J Clin Endocrinol Metab. 2006;91(10):3897‐3902. 16849410 10.1210/jc.2006-0558

[5] Simmonds MJ , Gough SC. Genetic insights into disease mechanisms of autoimmunity. Br Med Bull. 2005;71(1):93‐113. 15701924 10.1093/bmb/ldh032

[6] Rubtsov AV , Rubtsova K, Kappler JW, Marrack P. Genetic and hormonal factors in female-biased autoimmunity. Autoimmun Rev. 2010;9(7):494‐498. 20144912 10.1016/j.autrev.2010.02.008 PMC3171140

[7] Bakalov VK , Gutin L, Cheng CM, et al Autoimmune disorders in women with turner syndrome and women with karyotypically normal primary ovarian insufficiency. J Autoimmun. 2012;38(4):315‐321. 22342295 10.1016/j.jaut.2012.01.015 PMC3358475

[8] Radetti G , Mazzanti L, Paganini C, et al Frequency, clinical and laboratory features of thyroiditis in girls with Turner's Syndrome. Acta Pædiatr. 1995;84(8):909‐912. 10.1111/j.1651-2227.1995.tb13791.x 7488816

[9] Elsheikh M , Wass JA, Conway GS. Autoimmune thyroid syndrome in women with Turner's Syndrome–the association with karyotype. Clin Endocrinol. 2001;55(2):223‐226. 10.1046/j.1365-2265.2001.01296.x 11531929

[10] El-Mansoury M , Bryman I, Berntorp K, Hanson C, Wilhelmsen L, Landin-Wilhelmsen K. Hypothyroidism is common in turner syndrome: results of a five-year follow-up. J Clin Endocrinol Metab. 2005;90(4):2131‐2135. 15623818 10.1210/jc.2004-1262

[11] Mortensen KH , Cleemann L, Hjerrild BE, et al Increased prevalence of autoimmunity in turner syndrome–influence of age. Clin Exp Immunol. 2009;156(2):205‐210. 19298606 10.1111/j.1365-2249.2009.03895.x PMC2759466

[12] Witkowska-Sędek E , Borowiec A, Kucharska A, et al Thyroid autoimmunity in girls with turner syndrome. Med Biol. 2017;1022:71‐76. 10.1007/5584_2017_42 28456931

[13] De Sanctis V , Khater D. Autoimmune diseases in Turner syndrome: an overview. Acta Biomed. 2019;90(3):341‐344. 31580326 10.23750/abm.v90i3.8737 PMC7233727

[14] Livadas S , Xekouki P, Fouka F, et al Prevalence of thyroid dysfunction in Turner's syndrome: a long-term follow-up study and brief literature review. Thyroid. 2005;15(9):1061‐1066. 16187915 10.1089/thy.2005.15.1061

[15] Wooten N , Bakalov VK, Hill S, Bondy CA. Reduced abdominal adiposity and improved glucose tolerance in growth hormone-treated girls with Turner syndrome. J Clin Endocrinol Metab. 2008;93(6):2109‐2114. 18349057 10.1210/jc.2007-2266 PMC2435647

[16] El-Mansoury M , Berntorp K, Bryman I, et al Elevated liver enzymes in Turner syndrome during a 5-year follow-up study. Clin Endocrinol. 2008;68(3):485‐490. 10.1111/j.1365-2265.2007.03166.x 18167134

[17] Frost AR , Band MM, Conway GS. Serological screening for coeliac disease in adults with Turner's syndrome: prevalence and clinical significance of endomysium antibody positivity. Eur J Endocrinol. 2009;160(4):675‐679. 19208776 10.1530/EJE-08-0846

[18] Mårild K , Størdal K, Hagman A, Ludvigsson JF. Turner syndrome and celiac disease: a case-control study. Pediatrics. 2016;137(2):e20152232. 10.1542/peds.2015-2232 26746404

[19] Wilhelmsen L , Johansson S, Rosengren A, Wallin I, Dotevall A, Lappas G. Risk factors for cardiovascular disease during the period 1985-1995 in Goteborg, Sweden. The GOT-MONICA project. J Intern Med. 1997;242(3):199‐211. 9350164 10.1046/j.1365-2796.1997.00163.x

[20] Trimpou P , Lindahl A, Lindstedt G, Olerod G, Wilhelmsen L, Landin-Wilhelmsen K. Secular trends in sex hormones and fractures in men and women. Eur J Endocrinol. 2012;166(5):887‐895. 22307572 10.1530/EJE-11-0808

[21] Landin-Wilhelmsen K . www.internetmedicin.se, Turner syndrome 2022.

[22] Landin-Wilhelmsen K , Bryman I, Windh M, Wilhelmsen L. Osteoporosis and fractures in Turner syndrome-importance of growth promoting and oestrogen therapy. Clin Endocrinol. 1999;51(4):497‐502. 10.1046/j.1365-2265.1999.00841.x 10583318

[23] Ivarsson A , Myléus A, Norström F, et al Prevalence of childhood celiac disease and changes in infant feeding. Pediatrics. 2013;131(3):e687‐e694. 23420914 10.1542/peds.2012-1015

[24] Stenberg AE , Sylvén L, Hedstrand H, Kämpe O, Hultcrantz M. Absence of autoantibodies connected to autoimmune polyendocrine syndrome type I and II and Addison's Disease in girls and women with Turner syndrome. J Negat Results Biomed. 2007;6:10. 18088406 10.1186/1477-5751-6-10 PMC2242795

[25] Jørgensen KT , Rostgaard K, Bache I, et al Autoimmune diseases in women with Turner's Syndrome. Arthritis Rheum. 2010;62(3):658‐666. 20187158 10.1002/art.27270

[26] Rossouw JE , Anderson GL, Prentice RL, et al Risks and benefits of estrogen plus progestin in healthy postmenopausal women: principal results from the Women's Health initiative randomized controlled trial. JAMA. 2002;288(3):321‐333. 12117397 10.1001/jama.288.3.321

[27] Adlersberg MA , Burrow GN. Focus on primary care. Thyroid function and dysfunction in women. Obstet Gynecol Surv. 2002;57(Supplement):S1‐S7. 12074547 10.1097/00006254-200203001-00001

[28] Schmidt J , Dahlgren E, Bryman I, et al High androgen levels protect against hypothyroidism. Acta Obstet Gynecol Scand. 2017;96(1):39‐46. 27861716 10.1111/aogs.13054 PMC6680242

[29] Glynn N , Kenny H, Salim T, et al Alterations in thyroid hormone levels following growth hormone replacement exert complex biological effects. Endocr Pract. 2018;24(4):342‐350. 29658834 10.4158/EP-2017-0223

[30] Kalantaridou SN , Calis KA, Vanderhoof VH, et al Testosterone deficiency in young women with 46XX spontaneous premature ovarian failure. Fertil Steril. 2006;86(5):1475‐1482. 17070197 10.1016/j.fertnstert.2006.04.028

[31] Saenger P , Wikland KA, Conway GS, et al Fifth international symposium on Turner syndrome. Recommendations for the diagnosis and management of Turner syndrome. J Clin Endocrinol Metab. 2001;86(7):3061‐3069. 11443168 10.1210/jcem.86.7.7683

[32] Gravholt CH , Andersen NH, Conway GS, et al Clinical practice guidelines for the care of girls and women with Turner syndrome: proceedings from the 2016 Cincinnati international Turner syndrome meeting. Eur J Endocrinol. 2017;177(3):G1‐G70. 28705803 10.1530/EJE-17-0430

[33] Al-Bluwi GSM , AlNababteh AH, Östlundh L, Al-Shamsi S, Al-Rifai RH. Prevalence of celiac disease in patients with Turner syndrome: systematic review and meta-analysis. Front Med (Lausanne). 2021;8:674896. 34222285 10.3389/fmed.2021.674896 PMC8247446

